# Voluntary vs Electrically Stimulated Activation in Age-Related Muscle Weakness

**DOI:** 10.1001/jamanetworkopen.2019.12052

**Published:** 2019-09-25

**Authors:** Brian C. Clark, Todd M. Manini, Nathan P. Wages, Janet E. Simon, Leatha A. Clark

**Affiliations:** 1 Ohio Musculoskeletal and Neurological Institute (OMNI), Ohio University, Athens; 2 Department of Aging and Geriatric Research, University of Florida, Gainesville

## Abstract

This cross-sectional study compares voluntary neural activation of lower extremity muscles in clinically weak older adults vs stronger older adults.

## Introduction

The scientific and medical communities have long recognized that muscle weakness is a major risk factor for physical limitations, outright physical disability, and early death in older adults.^1^ It was long assumed that age-related loss of lean mass (ie, sarcopenia) was the primary contributor to weakness. This led to a number of pharmaceutical companies pursuing compounds that mechanistically act on muscle (eg, hypertrophy-inducing myostatin inhibitors) with the goal being to enhance muscle and physical function.^2^ Most of these have failed to enhance muscle strength and physical function, in part because of the multiple factors associated with muscle strength and physical function that extend well beyond muscle mass. For instance, longitudinal data clearly demonstrate that loss of strength is only modestly associated with loss of mass in older adults.^3^ Given that muscle force production is driven both by skeletal muscle size and quality as well as the nervous system,^4^ this cross-sectional study specifically examined the role of the nervous system in clinically meaningful, age-related weakness. Specifically, we sought to determine whether older adults with clinically meaningful weakness exhibit impairments in their nervous systems’ (peripheral and central) ability to activate their lower extremity muscles compared with their stronger counterparts. Here, we calculated the degree of voluntary inactivation (VIA) by comparing voluntary and electrically stimulated muscle forces.^5^ While this does not give insight about where in the nervous system impairment may occur, it does provide insight into whether the nervous system may have a global involvement in weakness.

## Methods

The Ohio University institutional review board approved this cross-sectional study, and participants provided written informed consent. From July 2015 to October 2018, 67 community-dwelling older adults without overt neurological disorders (mean [SD] age, 75.2 [6.9] years; 44 [66%] women) performed maximal isokinetic leg extension strength tests (BioDex System 4)^6^ and were subsequently classified as weak, modestly weak, or strong based on strength, identifying older adults at high and low risk of developing severe mobility limitations.^6^ We quantified VIA using the doublet interpolation technique similar to our prior work by supramaximally electrically stimulating the quadriceps muscle group (DS7AH; Digitimer) during a maximal voluntary effort with any increment in force evoked by the stimulus indicating deficits in voluntary activation.^5^ Estimates of lean mass were obtained from a whole-body dual-energy radiographic absorptiometry scan (Discovery W; Hologic). Group differences were examined using analysis of variance with sex entered as a factor, and Sidak post hoc tests were conducted on any significant analyses of variance using SPSS statistical software, version 25 (IBM). Two-tailed *P* ≤ .05 was considered statistically significant.

## Results

Descriptive statistics are shown in the Table. In all, 19 participants were characterized as weak, 29 as modestly weak, and 18 as strong. Notably, the weak participants were older (mean [SD] age, 78.4 [7.1] years vs 74.9 [7.3] years for modestly weak and 72.0 [5.0] years for strong), weighed more (mean [SD] weight, 78.3 [14.3] kg vs 73.3 [15.6] kg for modestly weak and 65.2 [11.7] kg for strong), had a higher body mass index (calculated as weight in kilograms divided by height in meters squared) (mean [SD] body mass index, 29.9 [4.6] vs 26.9 [4.3] for modestly weak and 23.7 [3.4] for strong), and had lower levels of mobility, physical function, and physical activity than their stronger counterparts (for example, mean [SD] Short Physical Performance Battery score for weak participants, 10.2 [1.2] vs 11.2 [0.9] for modestly weak and 11.8 [0.5] for strong) (Table). With regard to VIA capacity, there was no group × sex interaction (*F*_2,60_ = 0.982; *P* = .38), but a group main effect was noted (*F*_2,60_ = 4.010; *P* = .02). Post hoc testing indicated that the weak older adults exhibited significantly higher levels of VIA than the strong older adults (14.2% vs 7.1%; difference, −7.1%; 95% CI, −12.3% to −1.9%; *P* = .008) (Figure).

**Table.** Descriptive Characteristics of the Participants

Characteristic	Mean (SD)		
	Strong (n = 18)	Modestly Weak (n = 29)	Weak (n = 19)
Age, y	72.0 (5.0)	74.9 (7.3)	78.4 (7.1)^a^
Women, No. (%)	12 (67)	21 (72)	12 (63)
Isokinetic strength, N-m/kg	1.7 (0.3)	1.2 (0.1)^b^	0.8 (0.2)^a^^,^^c^
Height, cm	165.7 (9.8)	164.6 (8.2)	161.7 (12.7)
Weight, kg	65.2 (11.7)	73.3 (15.6)	78.3 (14.3)^a^
BMI	23.7 (3.4)	26.9 (4.3)^b^	29.9 (4.6)^a^
Morbid obesity: BMI ≥35, No. (%)	0	1 (3)	2 (10)
Appendicular lean mass, kg/height, m^2^	6.6 (1.2)	6.5 (1.1)	6.9 (1.2)
Lean thigh mass, kg	4.7 (1.0)	4.6 (1.1)	4.8 (0.9)
Short Physical Performance Battery score	11.8 (0.5)	11.2 (0.9)^b^	10.2 (1.2)^a^
6-Minute walk gait speed, m/s	1.6 (0.2)	1.4 (0.2)^b^	1.1 (0.3)^a^^,^^c^
Moderate activity, min/wk	132.4 (41.7)	104.7 (56.0)	88.1 (52.2)^a^
Repeatable Battery for the Assessment of Neuropsychological Status score	107 (10.5)	109.8 (12.6)	101.9 (11.2)
Charlson Comorbidity Index score	3.8 (0.8)	4.0 (1.2)	4.3 (0.7)
Center for Epidemiological Studies Depression score	6.4 (3.8)	6.6 (5.9)	8.0 (7.4)
Knee Injury and Osteoarthritis Outcome score	89.3 (12.0)	91.7 (10.7)	80.5 (19.2)^c^
Quality of Life in Neurological Disorders scores			
Fatigue	28.4 (7.5)	28.0 (9.0)	35.3 (13.5)
Cognitive function	120.0 (12.9)	115.2 (21.2)	118.3 (10.5)
Sleep disturbance	14.8 (3.9)	13.1 (3.9)	15.4 (5.0)
Lower extremity function	92.1 (3.4)	89.3 (7.5)	79.9 (14.6)^a^^,^^c^
Upper extremity function	99.2 (1.2)	98.0 (2.2)	93.3 (13.6)^a^
Satisfaction with social roles and activities	37.1 (4.6)	37.3 (4.2)	35.2 (5.9)

Abbreviation: BMI, body mass index (calculated as weight in kilograms divided by height in meters squared).

^a^ Weak significantly different from strong (*P* ≤ .05).

^b^ Modestly weak significantly different from strong (*P* ≤ .05).

^c^ Weak significantly different from modestly weak (*P* ≤ .05).

**Figure.**
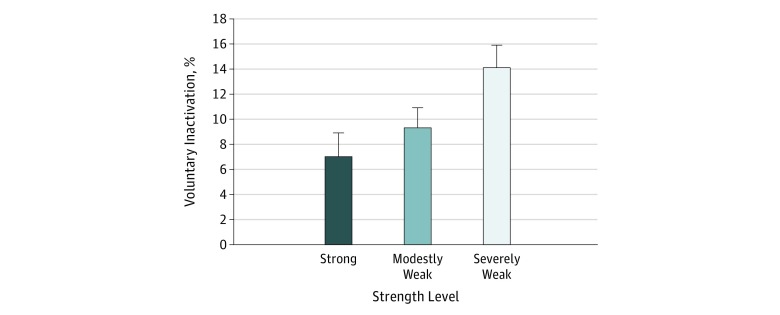
Voluntary Activation by Strength Level Weak participants experienced significantly higher mean levels of voluntary (neural) inactivation compared with strong participants (*P* = .008). A level of 0% indicates complete activation; error bars, standard error of the mean.

## Discussion

Weakness in older adults is conceptualized by many as a disorder of skeletal muscle. However, this work presents evidence indicating that weak older adults have significant deficits in their nervous systems’ ability to fully activate their leg extensor muscles. There are multiple locations in the neuromuscular pathway that could account for the deficits that were observed. For instance, this deficit could be due to suboptimal descending drive due neurophysiological and/or motivational factors, the motor neurons themselves not optimally responding to the descending drive, or other factors. While these data do not point to the underlying mechanism, they do suggest that medical treatments targeting the nervous system could be used to enhance muscle strength to prevent future health risks in weak older adults.
